# Exogenous p53 and ASPP2 expression enhances rAdV-TK/GCV-induced death in hepatocellular carcinoma cells lacking functional p53

**DOI:** 10.18632/oncotarget.7749

**Published:** 2016-02-26

**Authors:** Xiuhong Liu, Shuang Wang, Xianghua Guo, Feili Wei, Jiming Yin, Yunjin Zang, Ning Li, Dexi Chen

**Affiliations:** ^1^ Beijing You'an Hospital Affiliated with Capital Medical University, Beijing 100069, China; ^2^ Beijing Institute of Hepatology, Capital Medical University, Beijing 100069, China

**Keywords:** gene therapy, p53, ASPP2, ganciclovir, hepatocellular carcinoma

## Abstract

Suicide gene therapy using herpes simplex virus-1 thymidine kinase (HSV-TK) in combination with ganciclovir (GCV) has emerged as a potential new method for treating cancer. We hypothesize that the efficacy of HSV-TK/GCV therapy is at least partially dependent on p53 status in hepatocellular carcinoma (HCC) patients. Using recombinant adenoviral vectors (rAdV), TK, p53, and ASPP2 were overexpressed individually and in combination in Hep3B (p53 null) and HepG2 (p53 wild-type) cell lines and in primary HCC tumor cells. p53 overexpression induced death in Hep3B cells, but not HepG2 cells. ASPP2 overexpression increased rAdV-TK/GCV-induced HepG2 cell death by interacting with endogenous p53. Similarly, ASPP2 reduced survival in rAdV-TK/GCV-treated primary HCC cells expressing p53 wild-type but not a p53 R249S mutant. Mutated p53 was unable to bind to ASPP2, suggesting that the increase in rAdV-TK/GCV-induced cell death resulting from ASPP2 overexpression was dependent on its interaction with p53. Additionally, γ-H2AX foci, ATM phosphorylation, Bax, and p21 expression increased in rAdV-TK/GCV-treated HepG2 cells as compared to Hep3B cells. This suggests that the combined use of HSV-TK, GCV, rAdV-p53 and rAdV-ASPP2 may improve therapeutic efficacy in HCC patients lacking functional p53.

## INTRODUCTION

Surgical resection remains the most effective therapy for hepatocellular carcinoma (HCC); however, less than 15% of patients benefit from this treatment due to the presence of multiple tumor nodules. Other methods used to treat HCC, including radiofrequency ablation (RFA), microwave therapy, percutaneous ethanol injection (PEIT), radiotherapy, and biological radiotherapy, also have limited success. Gene therapy, including suicide gene therapy using herpes simplex virus-1 thymidine kinase (HSV-TK) in combination with the guanosine analog ganciclovir (GCV), has emerged as a potential new method for treating cancers.

The thymidine kinase (TK) gene product is involved in drug sensitivity, and plays a key role in DNA synthesis. TK derived from HSV-1 can phosphorylate the prodrug GCV into monophosphate GCV (GCV-MP). GCV-MP is similar in structure to deoxyguanosine triphosphate (dGTP), and its insertion into DNA sequences during replication inhibits DNA polymerase activity. The resulting single-stranded DNA breaks, leading to cell death [4]. Combined HSV-TK and GCV treatment has been studied in a variety of cancer types, including tumors of the brain, head and neck, skin, lung, liver, pancreas, colon, prostate, ovary, and breast. However, the results of several phase I and II clinical trials showed only slightly prolonged survival times [5, 6], possibly as a result of p53 mutation.

The p53 pathway mediates many cellular processes, including apoptosis, cell cycle regulation, and DNA repair [7, 8]. p53 function can be disrupted by TP53 gene mutations, alteration of upstream activating pathways, or alterations in downstream components that mediate p53 activity [9]. Mutations of the TP53 tumor suppressor gene have been reported in 23-67% of HCC patients worldwide and in 50% of HCC patients in China and South Africa [10–12]. Most of these mutations were found in the DNA binding region, which could prevent activation of target gene expression. In these cases, p53-mediated cell cycle arrest and apoptosis following HSV-TK/GCV-induced DNA damage may not occur [13, 14].

Apoptosis-stimulating of p53 protein 2 (ASPP2) can promote cell apoptosis by enhancing binding of p53 to the promoters of the pro-apoptotic genes BAX and PIG-3, upregulating their expression [15]. ASPP2 can also activate the cell cycle regulation target gene p21 for p53-mediated cell cycle inhibition. Compared to wild-type mice, ASPP2 heterozygous knockout mice were more susceptible to tumor formation [16]. Low levels of ASPP2 are correlated with poor prognosis in diffuse large B-cell lymphoma patients. Similarly, low ASPP2 expression is associated with resistance to genotoxic damage in lung cancer cell lines [17].

In the current study, two human liver cell lines with either wild type or null p53 status, and four primary HCC cell lines with either wild type or mutant p53, were treated with a recombinant adenoviral vector carrying TK in combination with the prodrugs GCV (rAdV-TK/GCV), rAdV-p53, and rAdV-ASPP2, either individually or in combination, in order to better understand the therapeutic mechanisms of HSV-TK/GCV in HCC.

## RESULTS

### rAdV-TK/GCV treatment induces death in HepG2 cells but not in Hep3B cells

**Figure 1:**
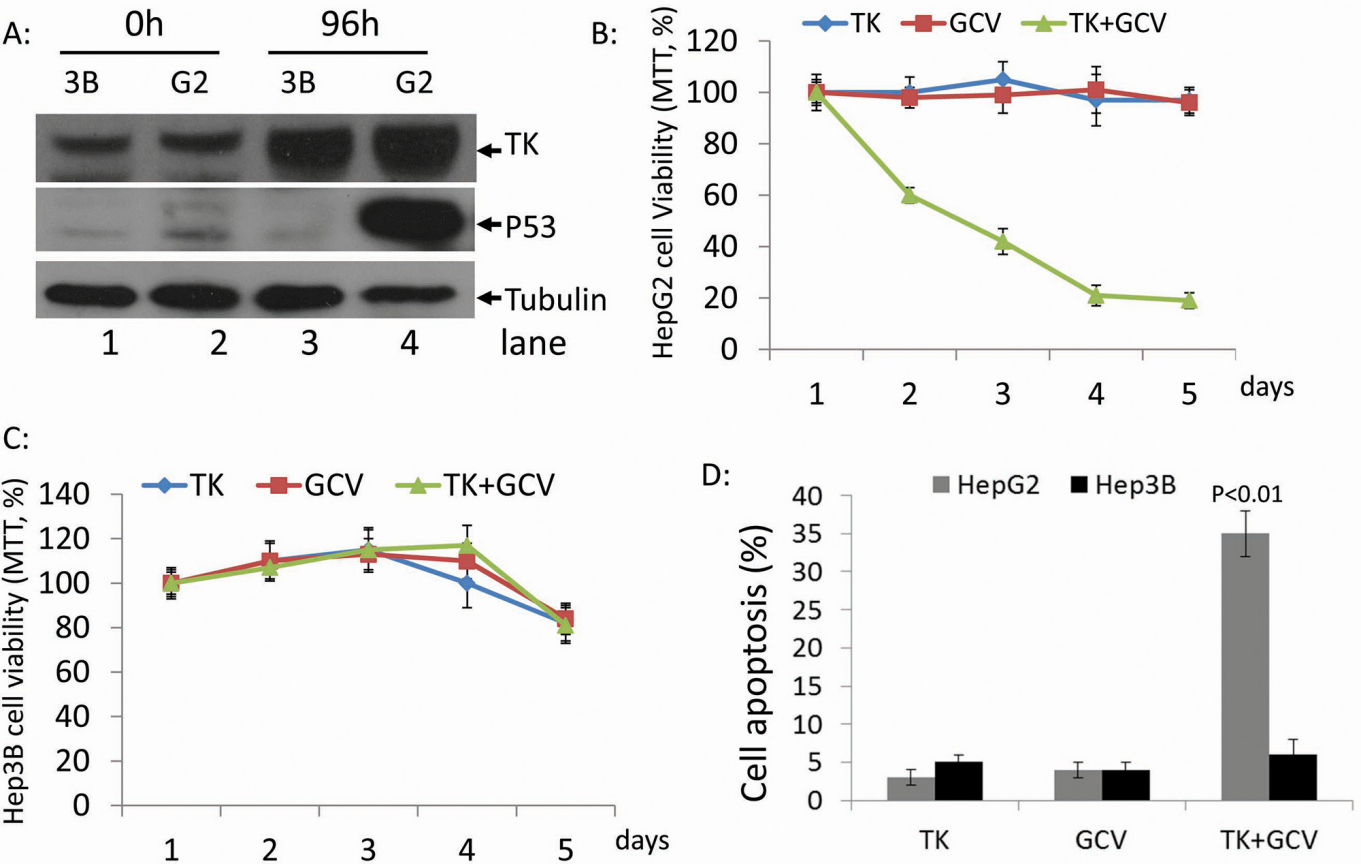
Effect of rAdV-TK/GCV on HepG2 cells (p53 wild-type) and Hep3B cells (p53 null) **A.** Western blotting was used to evaluate the levels of TK and p53 in HepG2 and Hep3B cells at 0 and 96 hours. The survival rates of **B.** HepG2 and **C.** Hep3B cells following rAdV-TK and GCV treatment for 1 to 5 days, as measured by MTT assay. **D.** Apoptosis analysis of HepG2 and Hep3B cells following rAdV-TK/GCV treatment on day 4.

We used the p53 null cell line, Hep3B, and the wild-type p53 cell line, HepG2, to study the effects of p53 status on rAdV-TK/GCV therapy in HCC. Treatment with rAdV-TK/GCV increased TK levels in both HepG2 and Hep3B cells, and p53 levels in HepG2 cells, over 48 hours (Figure 1A). Cell viability in both lines was unchanged after five days of treatment with either rAdV-TK or GCV alone (Figure 1B and 1C). Combined treatment with rAdV-TK and GCV led to a rapid decline in cell viability in HepG2 cells (Figure 1B), but not in Hep3B cells (Figure 1C), over the five-day treatment period. Treatment with both rAdV-TK and GCV also increased apoptosis in HepG2 cells more than rAdV-TK (*p* < 0.01) or GCV (*p* < 0.01) treatment alone. No difference in apoptosis was observed in Hep3B lines (*p* > 0.05).

### Overexpression of p53, but not ASPP2, causes death in Hep3B cells

**Figure 2:**
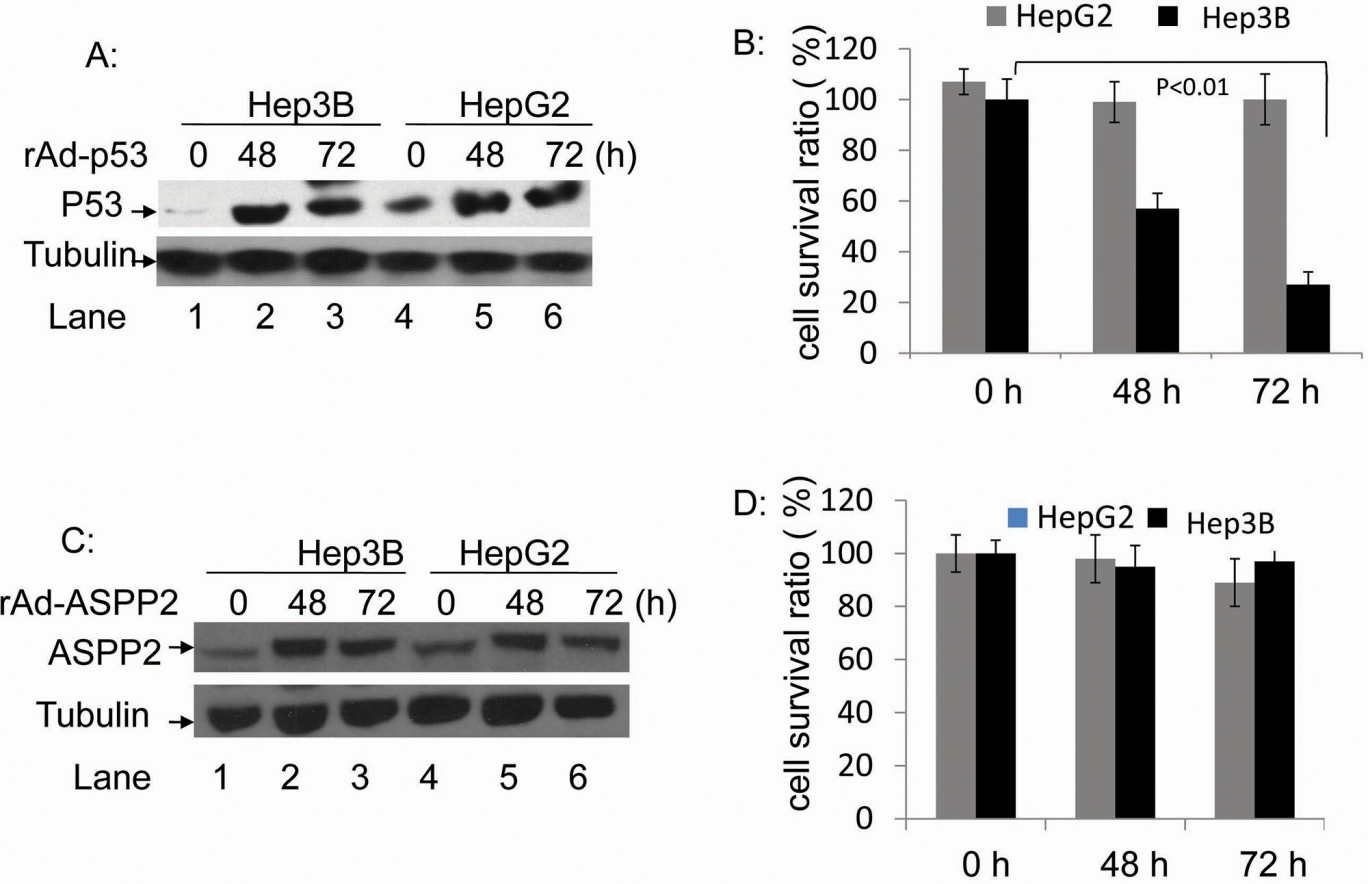
The rAdV-p53- and rAdV-ASPP2-induced cell death in HepG2 and Hep3B cells **A.** Western blotting analysis of p53 overexpression following rAdV-p53 infection and **C.** ASPP2 overexpression following rAdV-ASPP2 infection. **B.** MTT assay results of cell survival rate in HepG2 and Hep3B cells following rAdV-p53 infection and **D.** rAdV-ASPP2 infection.

HepG2 and Hep3B cells were infected with rAdV-p53 at 1×10^7^ copies/ml for 48 and 72 hours, and overexpression of p53 was confirmed by Western blotting (Figure 2A). The viabilities of HepG2 cells were 107.5%, 99.7% and 100.1%, at 0, 48, and 72 hours after infection, respectively; these differences were not statistically significant (*p* > 0.05) (Figure 2B). On the other hand, survival rates of rAdV-p53-infected Hep3B cells decreased from 91.3% to 57.9% and 27.7% at the same time points (*p* < 0.01) (Figure 2B). Lentivirus-siRNA P53 was then used to reduce endogenous p53 levels in HepG2 cells and found that HepG2-p53 RNAi cells treated with rAdV-TK/GCV alone exhibited more viability than HepG2-p53 RNAi cells treated with rAdV-p53 and rAdV-TK/GCV (Supplementary Figure S1A and S1B). HepG2 and Hep3B cells were then treated with rAdV-ASPP2 (1×10^7^ copies/ml); ASPP2 overexpression at 48 and 72 hours is shown in Figure 2C. Survival rates of HepG2 cells were 107.5%, 99.7%, and 100.1%, and survival rates of Hep3B cells were 107.5%, 99.7% and 100.1% (*p* > 0.05) at 0, 48, and 72 hours after rAdV-ASPP2 infection, respectively (Figure 2D). Overexpression of ASPP2 alone, via rAdV-ASPP2 infection, did not impact cell viability.

### Overexpression of p53, but not ASPP2, increases rAdV-TK/GCV-induced death in Hep3B cells

**Figure 3:**
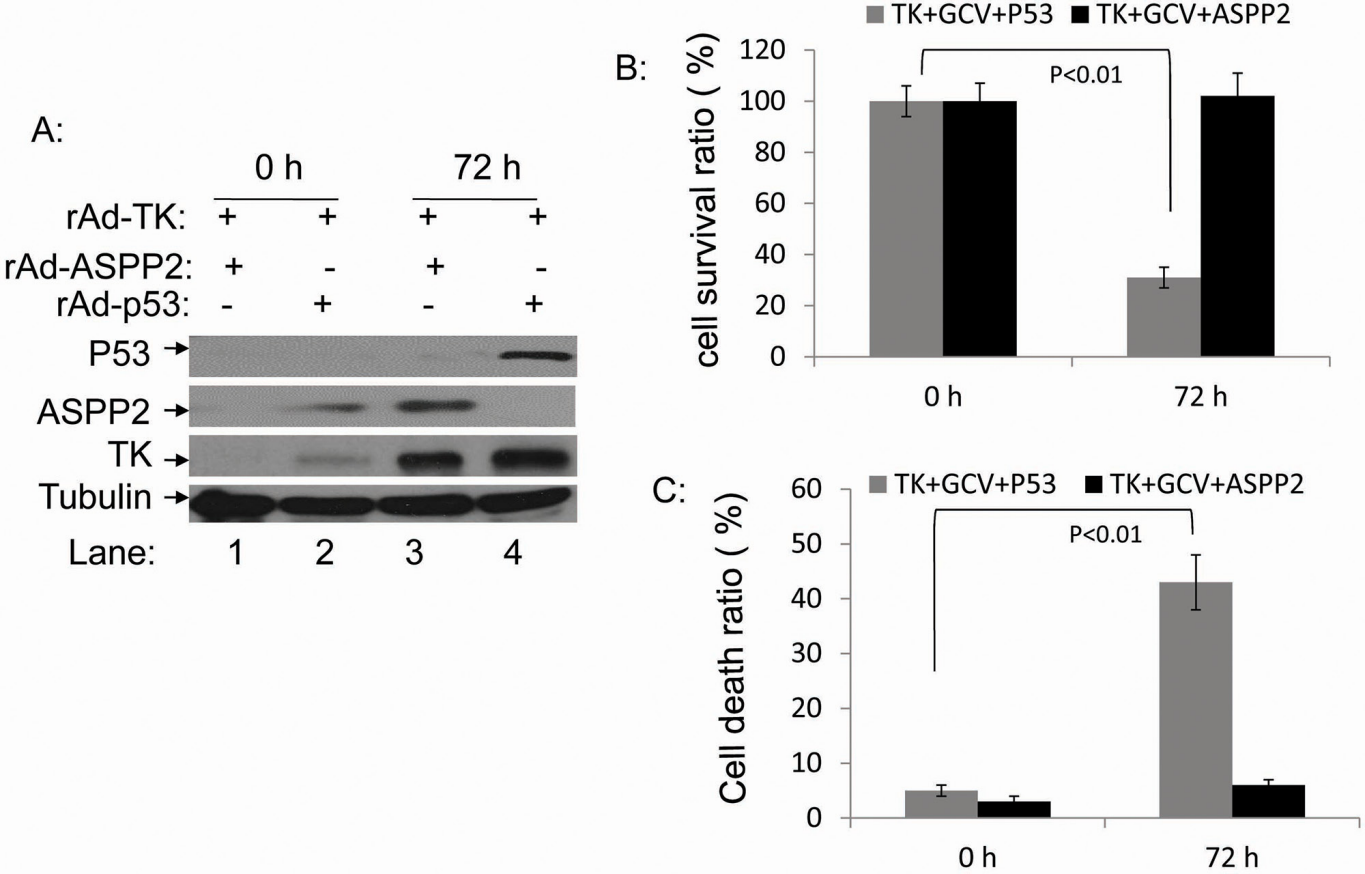
rAdV-p53 but not rAdV-ASPP2 increases rAdV-TK/GCV-induced death in Hep3B cells **A.** Expression levels of p53, ASPP2, and TK 0 and 72 hours after infection with rAdV-p53, rAdV-ASPP2, and rAdV-TK as detected by western blotting. **B.** Comparison of the survival ratios and **C.** death ratios in rAdV-TK/GCV-treated Hep3B cells 0 and 72 hours after infection with rAdV-p53 or rAdV-ASPP2.

Because rAdV-TK/GCV treatment failed to induce Hep3B cell death, we co-infected Hep3B cells with rAdV-p53 and rAdV-TK/GCV to determine whether p53 restoration altered the effect of rAdV-TK/GCV on cell death. In addition, we co-infected Hep3B lines with rAdV-p53, rAdV-ASPP2, and rAdV-TK/GCV for 72 hours. Expression of p53, ASPP2, and TK was confirmed by Western blotting (Figure 3A). HepB3 cell viability was analyzed using the MTT cell proliferation assay over four days. At four days, cell viability of the triple co-infected Hep3B cells was 31%; however, the survival rate for rAdV-ASPP2 and rAdV-TK/GCV co-infected Hep3B cells was 101% (Figure 3B). Apoptosis rates were 27% in rAdV-p53 and rAdV-TK/GCV co-infected lines and 5% in rAdV-ASPP2 and rAdV-TK/GCV co-infected cells (Figure 3C). These results suggest that p53, but not ASPP2, increases rAdV-TK/GCV cytotoxicity in the p53-null Hep3B cell line.

### rAdV-TK/GCV induces endogenous p53 expression via phosphorylation of ATM and γ-H2AX

**Figure 4:**
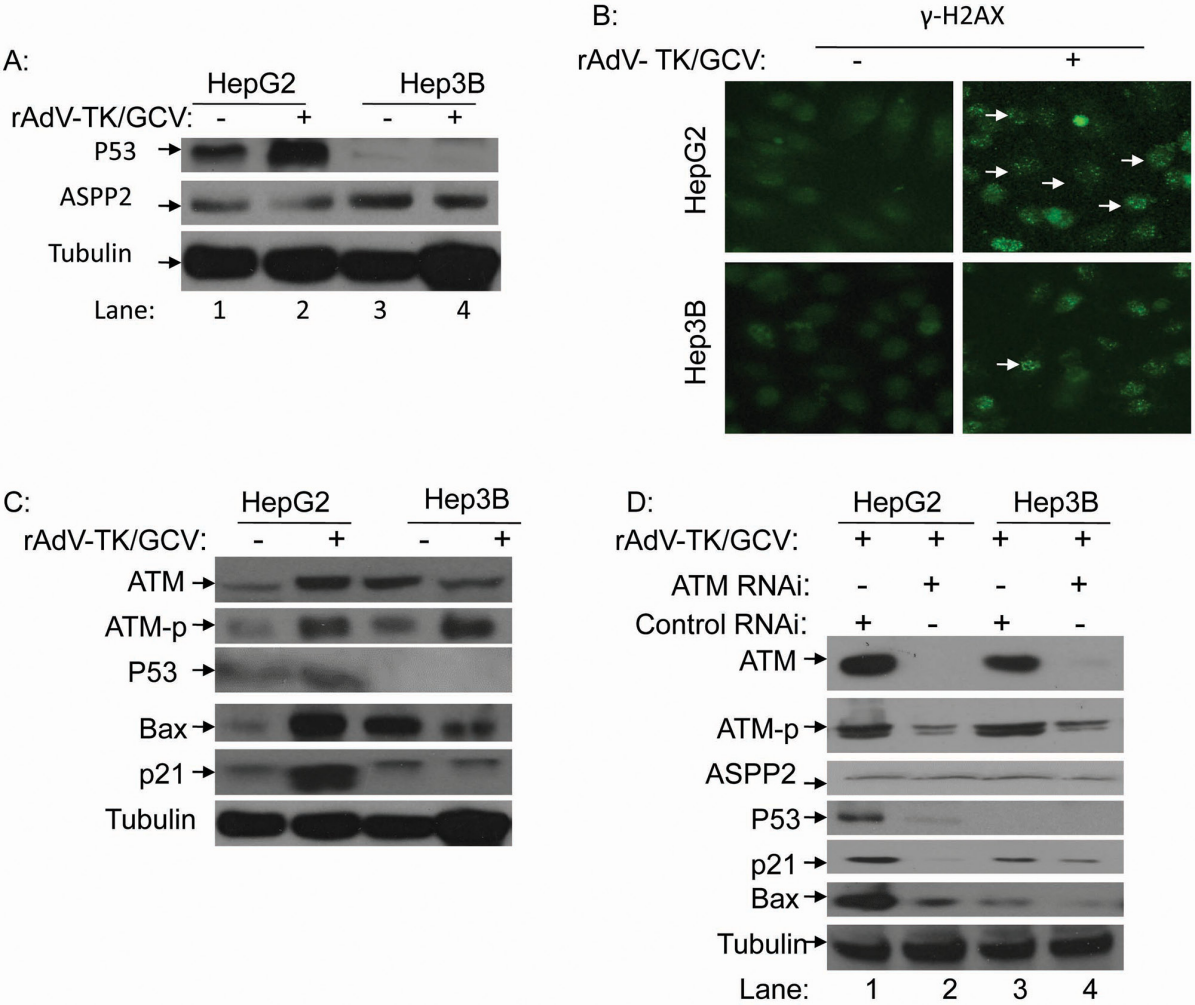
rAdV-TK/GCV treatment can induce endogenous p53 expression via phosphorylation of ATM and γ-H2AX **A.** Endogenous p53 and ASPP2 expression in HepG2 cells following rAdV-TK/GCV treatment. **B.** Analysis of γ-H2AX foci formation by fluorescence microscopy in HepG2 and Hep3B cells with or without rAdV-TK/GCV treatment. **C.** Western blot analysis of ATM, ATM-p, Bax, and p21 expression following rAdV-TK/GCV treatment for 48 hours. **D.** Effect of ATM RNAi on ASPP2, p53, p21, and Bax expression in HepG2 cells. (ATM-p: phosphorylation of ATM).

We found that rAdV-TK/GCV treatment induced HepG2 cell cycle arrest (Supplementary Figure S3) and apoptosis (Figure 1 and 3); exogenous overexpression of p53 via rAdV-p53 infection did not change this effect. Western blot results showed that p53 levels were higher in HepG2 cells treated with rAdV-TK/GCV for 48 hours, but ASPP2 levels were not affected in either HepG2 or Hep3B cells (Figure 4A). Further testing showed that p53-upregulated p21 and Bax levels in HepG2 cells were higher following rAdV-TK/GCV treatment for 48 hours. However, p21 and Bax expression in Hep3B cells did not change under the same conditions (Figure 4B and 4C).

More γ-H2AX foci were detected in HepG2 cells following rAdV-TK/GCV treatment (Figure 4B). Additionally, ATM phosphorylation levels increased in both HepG2 and Hep3B cells following treatment (Figure 4C). Following RNAi inhibition of ATM, p53 and its target genes were downregulated in HepG2 cells (Figure 4D). These results suggest that p53 plays an important role in apoptosis induced by rAdV-TK/GCV, and endogenous p53 upregulation following treatment results in part from increased ATM expression and phosphorylation.

### p53 combined with ASPP2 promotes rAdV-TK/GCV-induced death in Hep3B cells

**Figure 5:**
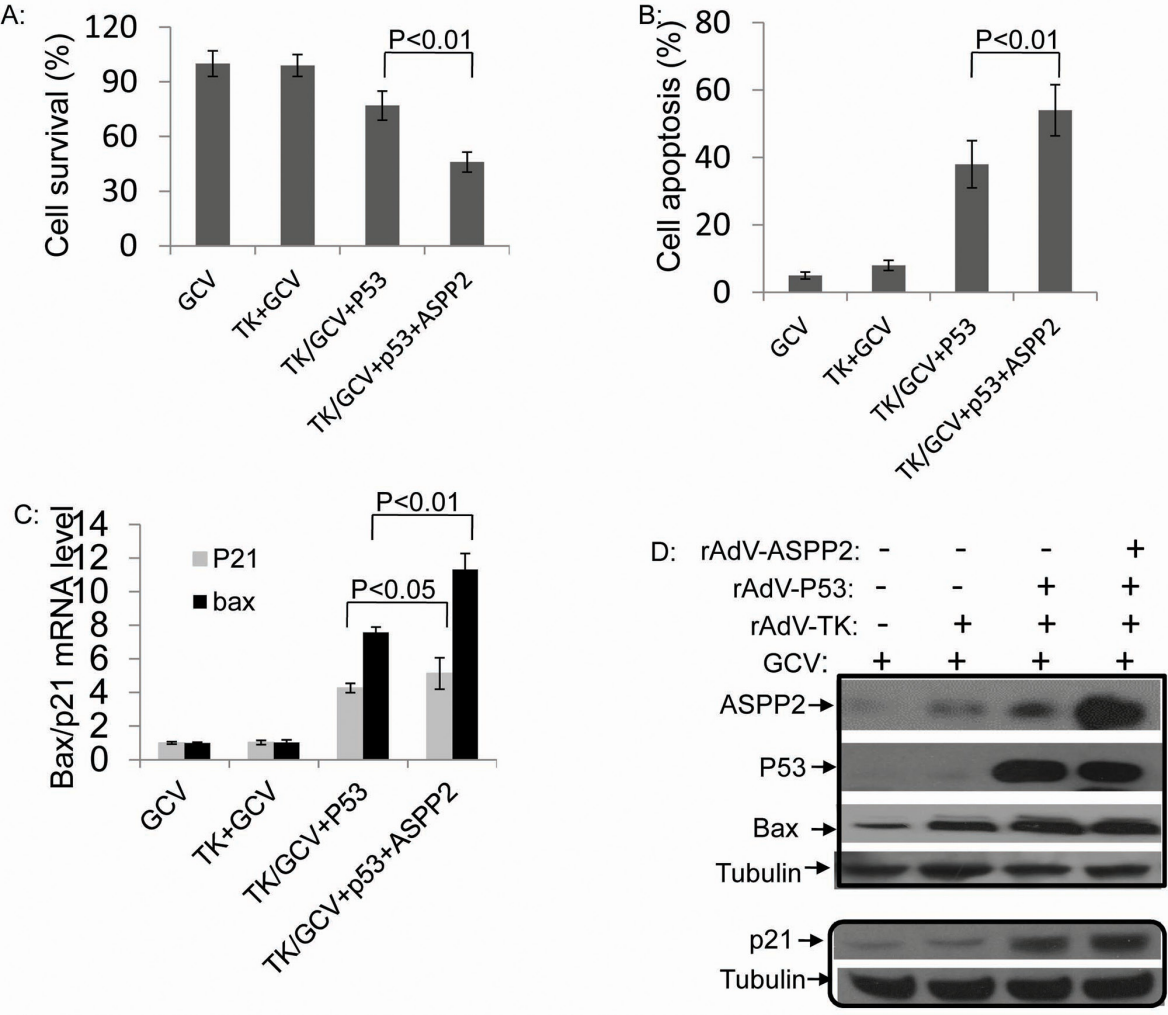
Combining p53 with ASPP2 promotes rAdV-TK/GCV-induced death in Hep3B (p53 null) cells **A.** Cell survival rate and **B.** apoptosis rate in Hep3B cells with rAdV-p53, rAdV-ASPP2, and rAdV-TK/GCV. **C.** mRNA levels of p21 and Bax after treatment with GCV alone, rAdV-TK/GCV, rAdV-p53 and rAdV-TK/GCV, or rAdV-P53, rAdV-ASPP2, and rAdV-TK/GCV treatment. **D.** Protein levels shown by Western blotting assay.

To study the effects of ASPP2 and p53 co-overexpression on p53-null HCC cells, Hep3B cells were co-infected with rAdV-ASPP2, rAdV-P53, and rAdV-TK/GCV. Compared to rAdV-p53 infection alone, co-infected cells exhibited increased cell death as detected by MTT assay. Early apoptosis detection with AnnexinV showed that ASPP2 overexpression increased rAdV-p53 and rAdV-TK/GCV-induced cell death (Figure 5A and 5B). The Hep3B survival rates detected by MTT assay were 73% with rAdV-p53 and rAdV-TK/GCV, and 45% following rAdV-p53, rAdV-ASPP2 and rAdV-TK/GCV co-infection.

ASPP2 overexpression also increased p21 and Bax expression at both the mRNA and protein levels, whereas GCV alone or rAdV-TK/GCV alone did not (Figure 5C and 5D).

### ASPP2 and p53 overexpression can enhance the therapeutic effect of rAdV-TK/GCV in primary HCC cultures

**Figure 6:**
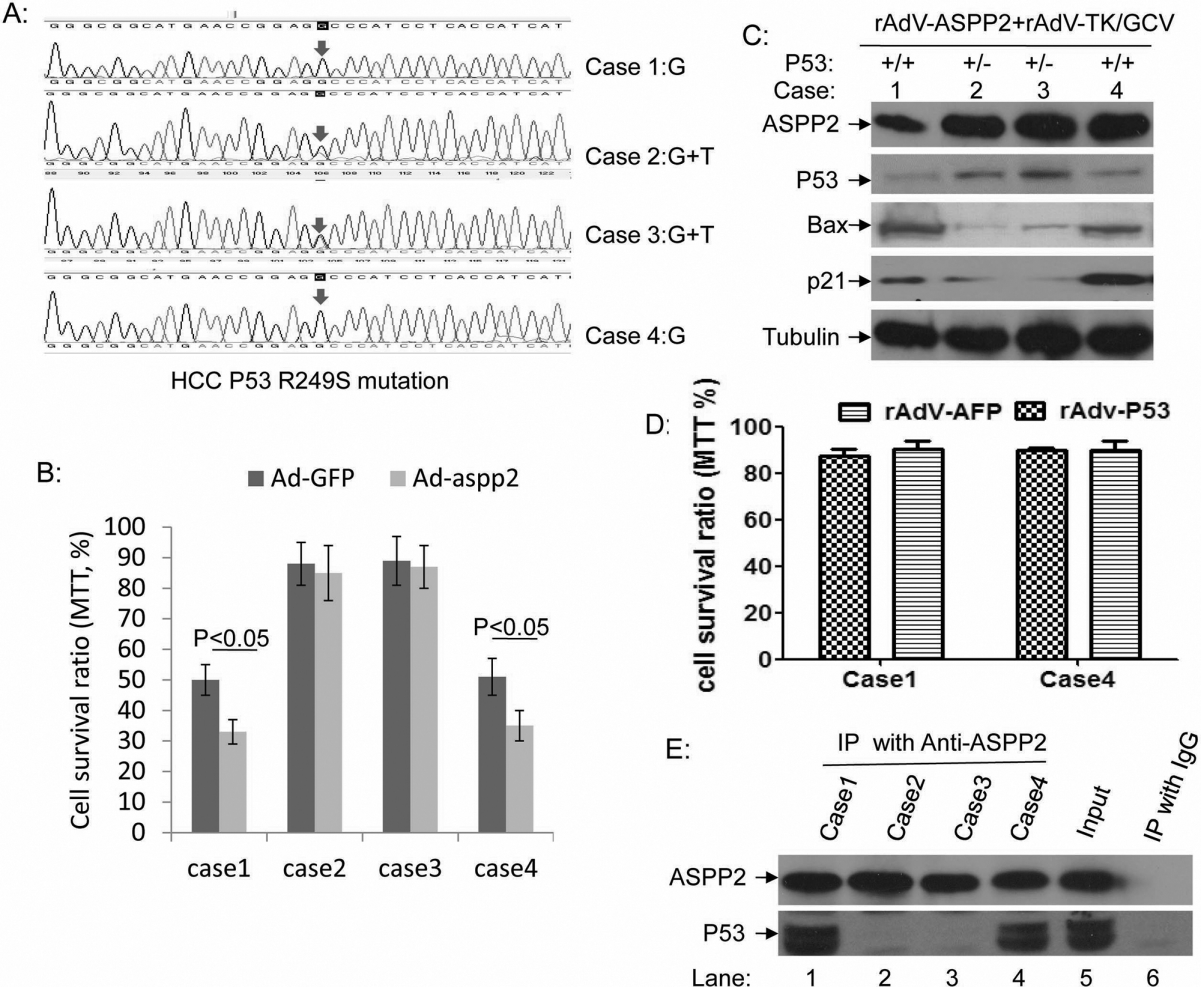
ASPP2 can enhance the therapeutic effect of rAdV-TK/GCV on primary cultured wild-type p53 HCC cells **A.** p53 gene sequences in four HCC cases, including two p53 wild-type primary HCC cells (cases 1 and 4) and two p53 R249S mutant primary HCC cells (cases 2 and 3). **B.** Comparison of rAdV-GFP- and rAdV-ASPP2-induced death in rAdV-TK/GCV-treated primary HCC cells. **C.** Bax and p21 levels in rAdV-ASPP2 and rAdV-TK/GCV-treated primary HCC cultures. **D.** Comparison of rAdV-GFP- and rAdV-p53-induced death in P53 wild-type primary HCC cells (cases 1 and 4). **E.** Co-immunoprecipitation results confirm that ASPP2 binds with p53 after rAdV-TK/GCV treatment in cases 1 and 4, but not in cases 2 and 3.

Most HCC-associated p53 mutations have been reported at p53 R249S. The p53 statuses of four fresh liver tissue samples excised from individual HCC patients at Beijing Youan Hospital were examined by PCR and DNA sequencing. Two cases included the p53 R249S mutation (cases 2 and 3), and two cases had wild-type p53 (cases 1 and 4) (Figure 6A). Primary tumor cells cultivated to the sixth generation were treated with rAdV-ASPP2 and rAdV-TK/GCV. rAdV-ASPP2 reduced survival of rAdV-TK/GCV-treated p53 wild-type primary HCC cells, but not of p53 R249S cells (Figure 6B). p53 knockdown primary wild-type p53 HCC cells had higher viability than parental primary HCC cells (case 1 and 4), when they treated with rAdV-ASPP2, and rAdV-TK/GCV (Supplementary Figure S2A and S2B). At the same time, only P53 R249S mutated primary HCC cells (case 2 and 3) were showed low cell viability after treatment with rAdV-TK/GCV, rAdV-P53 and rAdV-ASPP2 (Supplementary Figure S2C and S2D). These results further confirmed that endogenous P53 plays an important role in rAdV-p53, rAdV-ASPP2, and rAdV-TK/GCV-induced primary HCC death. Levels of the p53 target genes p21 and Bax were then measured in HCC cells. p21 and Bax levels increased in rAdV-ASPP2 and rAdV-TK/GCV-treated wild type p53 primary HCC cultures, but not in p53 R249S cells (Figure 6C). p53 overexpression in HepG2 cells, which have wild-type p53, did not affect cell death (Figure 2B); we further tested this effect in the p53 wild-type primary hepatocarcinoma cells (cases 1 and 4). No change in cell death was detected in the primary hepatocarcinoma cells following rAdV-P53 infection (Figure 6D). Co-immunoprecipitation results showed that ASPP2 can interact with rAdV-TK/GCV-induced p53, but ASPP2 failed to bind in cells with mutated p53 or in the absence of rAdV-TK/GCV treatment (Figure 6E and Supplementary Figure S2E).

## DISCUSSION

Suicide gene therapy using the HSV-TK/GCV system is a well-characterized tool used in cancer therapy [18–23]. However, this treatment has demonstrated little efficacy in HCC cases in clinical practice, mostly due to low targeting and absence of the ‘bystander effect’. One reason for this low efficacy could be the high incidence of p53 mutation in HCC, which is also associated with poor HCC prognosis [24]. Our HSV-TK/GCV phase III clinical trial found that some patients have good outcomes, but other patients are not sensitive to the treatment. The results from the current study support the hypothesis that the effect of HSV-TK/GCV on HCC cells is closely related to p53 status.

In the present study, we evaluated whether gene therapy with rAdV-ASPP2, rAdV-p53, and rAdV-TK/GCV is more effective than gene therapy with rAdV-TK/GCV alone for the induction of tumor death with and without functional p53. First, we found that rAdV-TK/GCV-treated p53 wild-type cells (HepG2) rapidly declined in number as a result of the treatment, whereas p53 null cells (Hep3B) were unaffected (Figure 1B and 1C). This strongly suggests that the effect of rAdV-TK/GCV treatment was closely related to p53 status in HCC cells. rAdV-p53 can effectively reduce Hep3B, but not HepG2, cell viability, and rAdV-ASPP2 alone had no impact on either cell line. However, reduced Hep3B survival following rAdV-TK/GCV treatment depended on both ASPP2 and endogenous p53 activity (Figure 5). Thus, our study implicates p53 in rAdV-TK/GCV-induced cell death, which is enhanced by ASPP2. rAdV-TK/GCV-induced death in HepG2 cells (with wild-type p53) was not enhanced by p53 overexpression, suggesting that the combination of HSV-TK/GCV and rAdV-p53 would benefit only those patients whose tumors do not express functional p53. Our results also showed that both γ-H2AX foci levels and ATM phosphorylation levels, which induced p53 and p53 target genes Bax and p21, were higher in HSV-TK/GCV-treated HepG2 cells than in Hep3B cells (Figure 4).

The comet assay and γ-H2AX foci experiments by Ladd et al. have shown that HSV-TK/GCV can cause DNA double-stranded breaks [25], which are frequently irreversible and result in cell death [7]. Such DNA damage can increase levels of ATM (ataxia telangiectasia mutated) and ATR (ATM-Rad3-related), both of which induce phosphorylation of serine 15 on p53; this prevents the interaction of MDM2 with p53 and reduces p53 ubiquitination and degradation [26, 27]. Previous studies also demonstrated that GCV administration increased γ-H2AX induction in a dose-dependent manner, and this increase was also p53-dependent. However, studies have shown that nearly half of HCC patients harbor p53 mutations [28].

In HBV-related HCC, the most common p53 mutation occurs at amino acid 249 (R249S). To further confirm the relationship between p53 mutation status and clinical outcomes of HSV-TK/GCV therapy, we treated cultured primary HCC cells with and without p53 mutations with HSV-TK/GCV and rAdV-ASPP2. The treatment only had an effect on primary HCC cells with wild type p53. Furthermore, mutated p53 was unable to bind with ASPP2, which confirmed that the pro-apoptotic function of HSV-TK/GCV treatment is dependent on the interaction between ASPP2 and p53. Our found supported that the combined use of HSV-TK, GCV, rAdV-p53 and rAdV-ASPP2 may improve therapeutic efficacy in HCC patients lacking functional p53.

## MATERIALS AND METHODS

### Cell culture and treatment

The human HepG2 and Hep3B hepatoblastoma cells were grown in Dulbecco's modified Eagle's medium (DMEM) supplemented with 10% fetal bovine serum (FBS) (Invitrogen, Carlsbad, CA). Cells seeded on glass cover slips were used for immunofluorescence assays. Human primary hepatoma cells (PHCs) were isolated from specimens obtained from patients undergoing hepatic resections. The specimens used in this study were obtained for pathological examination and isolation of DNA for PCR. The PCR products were extracted and sequenced by Biomed, Inc. (Beijing, China); therefore, there was no need for the patient to sign an informed consent form. PHCs were isolated from prewashed HCC tissues using a two-step collagenase perfusion and were cultivated within two layers of rat-tail collagen. All cells were seeded in 6- or 24-well plates for rAdV infection and GCV treatment.

### Recombinant adenoviral vectors and lentinvirus

A recombinant replication-defective adenoviral vector (CE1A deleted) containing the HSV-TK gene (rAdV-TK) under the transcriptional control of the Rous sarcoma virus long terminal repeat was used for gene delivery. It was produced at Cancer Research Center, Tongji Hospital, Tongji Medical College, following protocols detailed elsewhere [11, 12, 29]. Viral particles (vp/ml) were determined by spectrophotometric absorption and the purified adenovirus (5×10^11^ vp/ml) was stored in phosphate-buffered saline (PBS) containing 10% glycerol at −80°C. Before injection, adenovirus was diluted to the dose specified for each experimental group. rAdV-p53 (1×10^12^ vp/ml) was gifted from Saibainuo Biotech. Inc., Shenzheng, China. GCV (0.25 g/ml) was purchased from Sigma. rAdV-ASPP2 (5×10^11^ vp/ml) was produced by our lab and was stored at −20°C. The lentinvirus-P53 RNAi (1×10^8^ vp/ml) was ordered from Heyuan BioTech. Inc., Shanghai, China.

### Quantitative RT-PCR assay

An RNeasy Mini Kit (Qiagen, Hilden, Germany) was used to isolate the total RNA from cultured cells with or without treatments. The SuperScript III System for RT-PCR (Invitrogen, Carlsbad, CA) was used to synthesize first-strand cDNA. SyBR Green was added for the detection of dsDNA products during RT-PCR. Relative mRNA levels were normalized to β-actin. The primers for real time quantitative PCR were as follows: for p21: 5′-CAGGCTGAAGGGTCCCCAGGTGGA-3′ (forward) and 5′- GGATTAGGGCTTCCTCTTGGAGA-3′ (reverse); for Bax: GGGCTCACAAGTTAGAGA CAAGCCTGGGCG (forward) and CGCCCAGGCTTGTCT CTAACTTGTGAGCCC (reverse); for β-actin: 5′- GCCCTGAGGCACTCTTCCA-3′ (forward) and 5′- CGGATGTCCACGTCACACTT-3′ (reverse).

### Western blotting

Cell lysates were subjected to Western blot as previously described [15]. Briefly, total lysates were separated by 12% SDS-PAGE and transferred to PVDF filters. After blocking with 5% non-fat milk, the membranes were probed with primary antibodies (Monoclonal antibodies: Anti-P53 DO-1 from NeoMarkers, Anti-ASPP2 C-terminal DX 54.10 from Sigma, and all others, including Tubulin, Bax, P21, ATM, and horseradish peroxidase-conjugated secondary antibodies, from Santa Cruz, CA, USA). The blots were developed using an enhanced chemiluminescence kit (Pierce SuperSignal, Thermo Fisher Scientific Inc. Rockford, IL, USA) and were exposed onto X-ray films.

### Cell apoptosis assay

Flow cytometric analysis was performed using BD FACSCanto II (Becton Dickinson). HepG2 and Hep3B cells were plated 24 hours prior to transfection with rAdV-TK, rAdV-ASPP2, rAdV-P53, or rAdV-GFP at a concentration of 1×10^7^ PFU/ml (in rAdV-ASPP2 and rAdV-P53 co-treatment groups [Figure 5], the concentration of rAdV-P53 was 2.5×10^6^ PFU/ml). After treatment with GCV at different time points, the cells were washed twice with cold PBS, re-suspended in Annexin V binding buffer (SouthernBiotech), and analyzed by flow cytometry.

### Cell viability assay (MTT method)

The supernatants were collected to assess cell viability using the Vybrant MTT cell Proliferation Assay Kit from Life Technologies (Invitrogen Life Technologies). Briefly, 20 μl of MTT solution were added to each well and thoroughly mixed into the media. The cells were then continually incubated (37°C, 5% CO_2_) for 3 hours to allow the MTT to be metabolized. Finally, after cells were removed from the media and dissolved with 200 μl DMSO, cell viability was detected by reading optical density at 560 nm and subtracting background at 670 nm.

### Analysis of γ-H2AX foci formation by fluorescence microscopy

Cells were grown on chambered slides for 48 hours prior to rAdV-TK/GCV infection. After incubation with rAdV-TK/GCV, the cells were washed with PBS and then fixed and permeabilized with acetone:methanol (50:50 v/v) for 10 min. The fixed cells were then washed with PBS, blocked with 10% goat serum for 1 hour, incubated with γ-H2AX primary antibody (1:500 dilution; Novus NB-100-3B4) for 1 hour, washed, incubated with FITC conjugated goat anti-rabbit secondary antibody (1:300 dilution; Jackson Immunology Research, USA) for 1 hour, washed, and mounted with DAPI (Vector, USA). Images of representative cell populations were captured, and γ-H2AX foci were counted visually.

### Co-immunoprecipitation assay (CO-IP)

CO-IP was performed as described previously [15]. Briefly, cell lysates (500 μg of protein in 500 μl of RIPA lysis buffer) were pre-cleared using protein A/G PLUS-agarose beads (Santa Cruz Biotechnology) and were incubated at 4°C overnight with anti-ASPP2 antibody. The immunocomplexes were separated by incubation with protein A/G agarose beads and were resolved using SDS-PAGE. Western blot analysis was performed to detect the p53 protein.

### Cell cycle arrest assay

Cells were harvested and re-suspended in PBS buffer at 1-2 × 10^6^ cells/ml in a 15 ml polypropylene, V-bottomed tube. Cells were then fixed with cold 100% ethanol for 1 hour at 4°C. After two PBS washes, the cells were re-suspended in PI staining solution (20 μg/ml propidium iodide, 0.5 μg/ml RNase A in PBS) for 3 hr at 4°C. The samples were stored at 4°C until analysis by flow cytometry

## SUPPLEMENTARY FIGURES


