# Supplementary material for: Exogenous p53 and ASPP2 expression enhances rAdV-TK/GCV-induced death in hepatocellular carcinoma cells lacking functional p53
Source: Oncotarget. 2016 Feb 26;7(14):18896–905. doi: 10.18632/oncotarget.7749 (PMC4951338; doi:10.18632/oncotarget.7749)
Supplement: Supplementary file 1 [file oncotarget-07-18896-s001.pdf]

SUPPLEMENTARY FIGURES

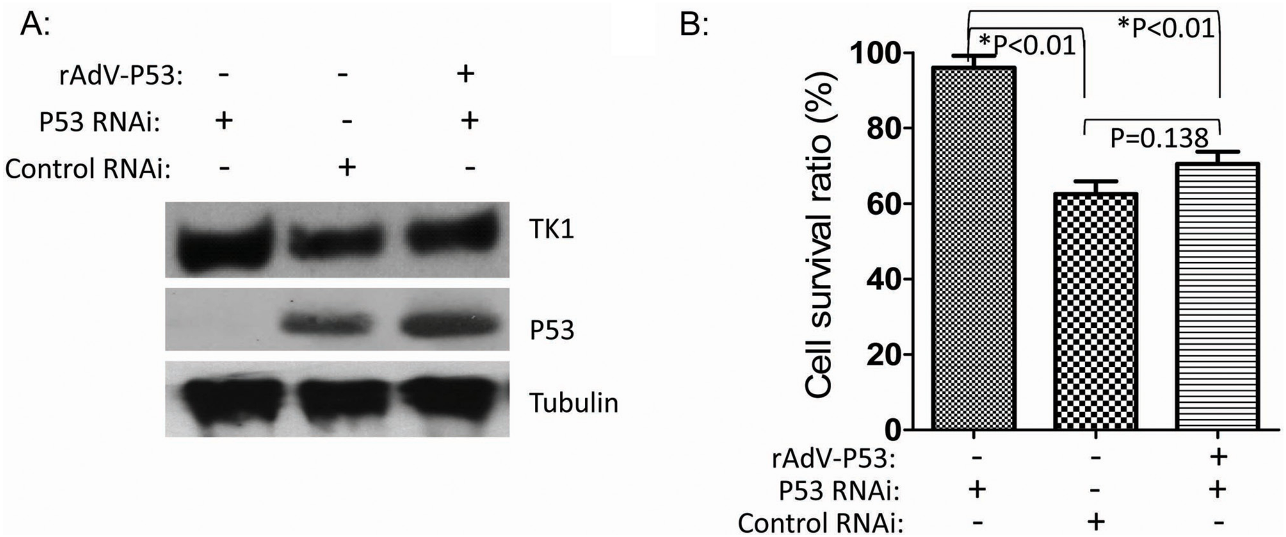

Supplementary Figure S1: The engineered p53 knockdown HepG2 is sensitive to rAdV-p53 and rAdV-TK/GCV induced cell death. A. Western blotting was used to evaluate the levels of TK and p53 in HepG2 following treatment with P53 RNAi, rAdV-P53 and rAdV-TK/GCV at 48 hours. **B.** The survival rates of HepG2 following P53 RNAi, Ad-P53 and AD-TK/GCV treatment over 72 hours, as measured by MTT assay.

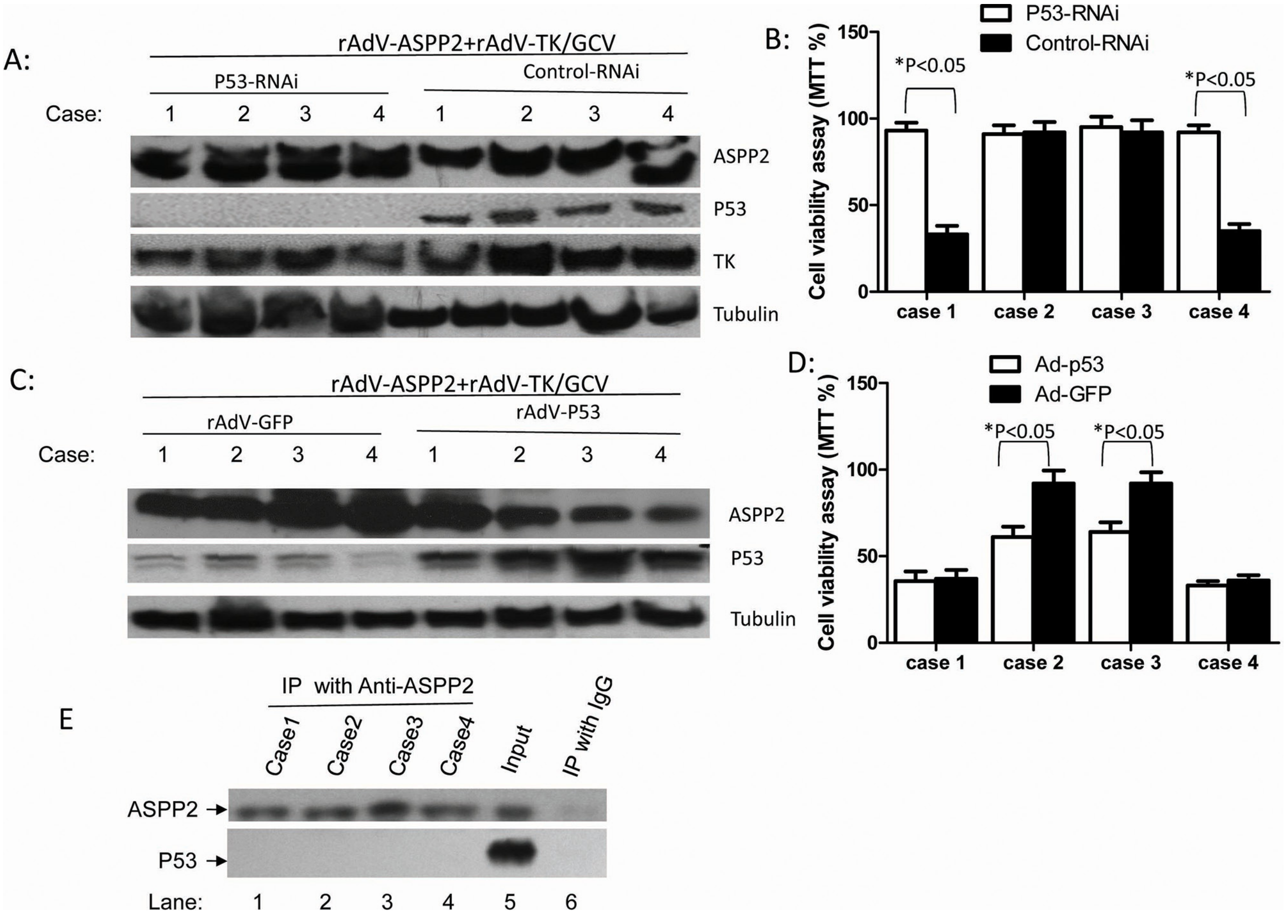

Supplementary Figure S2: The killing effects of rAdV-p53 and rAdV-ASPP2 in the primary hepatocarcinoma cells. A. Western blotting analysis of P53, ASPP2 and TK expression following Lentinvirus-P53 RNAi, rAdV-ASPP2 and rAdV-TK infection. **B.** MTT assay results of cell survival rate in the primary hepatocarcinoma cells with Lentinvirus-P53 RNAi, rAdV-ASPP2 and rAdV-TK infection. ASPP2 and TK/GCV induced cell death were mediated by wild type P53 in primary HCC ($P < 0.05$). **C.** Western blotting analysis of P53, ASPP2 expression following rAdV-P53, rAdV-ASPP2 and rAdV-TK infection. **D.** The combination of rAdV-p53 with rAdv-ASPP2 infection can promote the killing effect of rAdV-TK/GCV on p53N249A mutant primary HCC cells (cases 2 and 3, $P < 0.05$). **E.** Co-immunoprecipitation results showed that ASPP2 can't bind with p53 in no rAdV-TK/GCV treatment of the primary hepatocarcinoma cells.

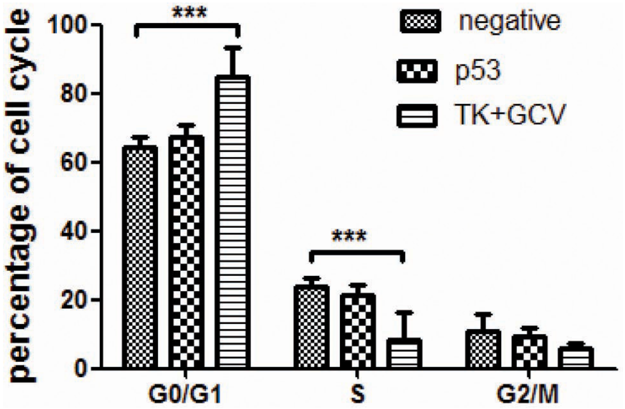

Supplementary Figure S3: HepG2 cells cycle arrest were induced by rAdV-TK/GCV but failed by rAdV-P53. ($P < 0.05$ in no any treatment (negative) via rAdV-TK/GCV treatment).
